# Prognostic Value of LC3A Protein Expression Patterns in Rectal Cancer Tumors

**DOI:** 10.3390/cancers17091568

**Published:** 2025-05-05

**Authors:** Vincent Ho, Liping Chung, Tristan Rutland, Vivienne Lea, Stephanie H. Lim, Askar Abubakar, Weng Ng, Mark Lee, Tara L. Roberts, Wei Chua, Scott Mackenzie, Cheok Soon Lee

**Affiliations:** 1 School of Medicine, Western Sydney University, Penrith, NSW 2751, Australia; liping.chung@westernsydney.edu.au (L.C.); t.rutland@westernsydney.edu.au (T.R.); vivienne.lea@health.nsw.gov.au (V.L.); askar.abubakar@westernsydney.edu.au (A.A.); tara.roberts@westernsydney.edu.au (T.L.R.); wei.chua@health.nsw.gov.au (W.C.); s.mackenzie@westernsydney.edu.au (S.M.); soon.lee@westernsydney.edu.au (C.S.L.); 2 Ingham Institute for Applied Medical Research, Liverpool, NSW 2170, Australia; stephanie.lim@health.nsw.gov.au; 3 Department of Anatomical Pathology, Liverpool Hospital, Liverpool, NSW 2170, Australia; 4 Department of Medical Oncology, Liverpool Hospital, Liverpool, NSW 2170, Australia; weng.ng@health.nsw.gov.au; 5 Macarthur Cancer Therapy Centre, Campbelltown Hospital, Campbelltown, NSW 2560, Australia; 6 Department of Radiation Oncology, Liverpool Hospital, Liverpool, NSW 2170, Australia; mark.lee2@health.nsw.gov.au; 7 South Western Sydney Clinical School, University of New South Wales, Liverpool Hospital, Liverpool, NSW 2170, Australia; 8 Discipline of Medical Oncology, School of Medicine, Western Sydney University, Liverpool, NSW 2170, Australia; 9 Department of Colorectal Surgery, Liverpool Hospital, Liverpool, NSW 2170, Australia; 10 Discipline of Pathology, School of Medicine, Western Sydney University, Campbelltown, NSW 2560, Australia

**Keywords:** rectal cancer, autophagy, microtubule-associated protein 1 light chain 3 alpha (LC3A), stone-like structures (SLS), tumor stage, prognosis, biomarker

## Abstract

**Background/Objectives**: Autophagy is a conserved self-degradation process by which cells break down and recycle their cellular constituents. This process is activated by various stressors, including nutrient deprivation and DNA damage, and has also been associated with tumor progression. In the present study, we aimed to determine the expression patterns, clinicopathological significance, and prognostic value of the autophagy marker microtubule-associated protein 1 light chain 3 alpha (LC3A)—an essential component of autophagic vacuoles—in rectal cancer. **Methods**: LC3A reactivity was measured by immunohistochemistry in tumor samples from 243 patients who underwent surgery for rectal cancer. **Results**: Three distinct patterns of LC3A expression were identified: diffuse cytoplasmic, perinuclear, and stone-like structures (SLS). In Kaplan–Meier survival analyses, patients positive for the SLS pattern of LC3A staining in the tumor periphery (TP) had worse overall survival (OS; *p* = 0.001) and disease-free survival (DFS; *p* = 0.030) than those without SLSs in this region, as determined by the log–rank test. When patients were stratified by tumor stage, this result was significant in those with stage T3–T4 (OS, *p* < 0.001; DFS, *p* = 0.001) but not T1–T2 disease. Multivariate Cox regression analysis further showed an association between TP-localized LC3A SLS positivity and reduced OS for the overall cohort (hazard ratio [HR] = 2.6313, 95% confidence interval [CI]: 1.090–6.349, *p* = 0.031) and specifically those in the T3–T4 subgroup (HR = 3.347, 95% CI: 1.657–6.760, *p* = 0.001). **Conclusions**: Our findings suggest that positivity for SLSs in the TP may hold clinical value as a biomarker for survival prognosis in rectal cancer patients.

## Simple Summary

Rectal cancers represent a growing proportion of colorectal cancer diagnoses, particularly in younger patients, and are associated with poor outcomes due to surgical challenges and an incomplete understanding of disease pathogenesis. Here, we investigated the relationship between rectal cancer outcomes and expression patterns of microtubule-associated protein 1 light chain 3 alpha (LC3A), a marker protein for the essential cellular self-degradation mechanism known as autophagy, in tumors from 243 rectal cancer patients. We found that a specific punctate pattern of LC3A staining in the tumor periphery, referred to as stone-like structures, was associated with worse overall survival and disease-free survival, particularly for patients with more advanced rectal cancer tumors (i.e., T3–T4). These findings suggest that the presence of “stone-like” formations comprising the autophagy marker LC3A at the peripheral regions of the tumor where metastasis is likely to begin is associated with rectal cancer malignancy and poor survival outcomes.

## 1. Introduction

Colorectal cancer (CRC) ranks among the deadliest malignancies, causing nearly 10% of all cancer-related deaths in the United States [1]. Concerningly, a growing proportion of new cases are occurring in younger individuals, a trend driven largely by left-sided/rectal tumors, with rectal cancer accounting for approximately 40% of CRC diagnoses in those aged 50–64 between 2015 and 2019 [2]. Outcomes for rectal cancer are also worse than for other CRCs, largely due to difficulties in resection resulting from their proximity to the mesorectal fascia and pelvic organs, as well as limited accessibility within the pelvic space [3].

Preoperative chemoradiotherapy followed by total mesorectal resection and adjuvant chemotherapy can reduce recurrence and improve outcomes for patients with rectal cancer [4]. However, response to treatment varies and long-term outcomes remain poor, particularly for those with locally advanced disease, prompting the exploration of alternative treatment regimens that have exhibited moderate success in some patient groups [4,5,6]. Consequently, significant effort has been directed at identifying biomarkers in rectal cancer for prognostic determination and predicting response to treatment [5]. Through these studies, we and others have identified numerous candidates, including genes involved in the DNA damage response, chemokine signaling, and the p53 pathway [5,6,7,8,9,10,11,12,13,14]. However, our understanding of the molecular and clinicopathological features that affect rectal cancer prognosis remains incomplete.

In addition to the pathways noted above, over the past two decades, the process of autophagy has been increasingly recognized as a key modulator of development, progression, and chemoresistance for multiple cancers, including that of the lung, breast, and pancreas [15,16,17,18,19,20]. Macroautophagy, commonly referred to as autophagy, is the essential, highly conserved catabolic process through which cells break down and recycle cellular components to prevent the accumulation of damaged organelles and maintain energy metabolism during states of nutrient deprivation [21,22]. Paradoxically, studies have shown that autophagy can function to suppress or promote oncogenesis, depending on the context [15,18,19]. For example, autophagy can inhibit cancer development by degrading oncogenic proteins and preventing accumulation of cancer-inducing reactive oxygen species from damaged organelles [20,22]. Conversely, once cancer has progressed, elevated levels of autophagy support the high metabolic needs of cancer cells, promote their survival under hypoxic conditions, and enhance treatment resistance [17,18,19,23]. The complex cross-talk between autophagy and apoptosis may further inhibit or induce death of malignant cells, depending on cellular context and upstream signaling [18,24]. Notably, autophagy pathways can also promote cancer cell survival in response to treatment, and accordingly, autophagy inhibition was shown to increase tumor cell sensitivity to chemoradiotherapy and other anti-cancer agents for multiple malignancies, including CRC [23,25].

Mutations in several autophagy genes, including autophagy-related gene 5 (*ATG5)*, UV irradiation resistance-associated gene (*UVRAG*), and *KRAS*, are associated with CRC development, suggesting a pivotal role for autophagy in this disease [26,27,28]. However, as for other cancers, the precise role of autophagy in CRC appears to vary by context, with prior studies reporting conflicting findings regarding the expression and clinical outcomes associated with various autophagy proteins, such as ATG5, Beclin-1, and the autophagosomal marker microtubule-associated protein 1 light chain 3 (LC3) [29,30]. In an attempt to clarify these findings, recent meta-analyses found that elevated LC3 expression is associated with improved survival in CRC patients, whereas increased levels of Beclin-1 are associated with poor survival outcomes [31,32,33]. Notably, however, prior investigations have reported differential associations between CRC outcomes and distinct LC3 staining patterns. For example, one study noted that stone-like structures (SLSs), a punctate pattern of LC3 alpha (LC3A) staining indicative of aberrant or excessive autophagy, was associated with metastasis and poor prognosis in CRC [34]. However, this study did not differentiate between cancers of the colon and rectum.

Here, to further assess the prognostic significance of the LC3 protein in rectal cancer, we measured the expression patterns of LC3A staining in central and peripheral tumor samples, as well as adjacent healthy tissue, from 243 patients. Our results reveal that the SLS pattern of LC3A staining within the peripheral regions (but not the center) of the tumor was associated with worse survival outcomes, specifically in patients with advanced disease (T3–T4). These findings suggest that the presence of tumor-peripheral SLS LC3A staining in rectal cancer may be indicative of more aggressive disease. Our results also imply that understanding the prognostic implications of LC3A (and potentially other autophagy proteins) in rectal cancer is dependent on determining the specific pattern and location of expression within tumor tissue. In the case of rectal cancer, the LC3A-SLS status may be a stratification tool for therapeutic decision-making contingent on prognostic and predictive data such as chemoradiotherapy resistance.

## 2. Materials and Methods

### 2.1. Sample Selection

This study included 243 tumor samples from patients diagnosed with stage I–IV rectal cancer between 2000 and 2011, all of which were histologically confirmed. The research was approved by the Human Research Ethics Committee (HREC) of the South Western Sydney Local Health District (HREC Reference: HREC/14/LPOOL/186; project number 14/103) in Sydney, Australia. Patients received one of two treatment regimens: a single 25-Gy dose given over five fractions or a total of 50.4 Gy delivered in 28 fractions together with 5-fluorouracil-based chemotherapy. Surgical interventions comprised total mesorectal excision, as well as anterior or abdominoperineal resection. Follow-up care included clinic visits, blood examinations, colonoscopies, and imaging, as advised by the treating specialist. The patient characteristics are outlined in Table 1.

### 2.2. Sample Preparation and Tissue Microarrays

Five sites were used to obtain tissue samples: the damaged lymph nodes (LN), the surrounding normal mucosa close to the tumor (NCT), the normal mucosa away from the tumor (NAT), the tumor center (TC), and the tumor periphery (TP) at the invasive boundary. Two cores, each 1 mm in diameter, were then removed from the formalin-fixed, paraffin-embedded (FFPE) tumor tissues. A Beecher Manual Tissue Microarrayer (Beecher Instruments Inc., Sun Prairie, WI, USA) was then used to insert these cores into pre-drilled wells of tissue microarray blocks, which were later put on slides for immunohistochemical examination.

### 2.3. Immunohistochemistry

LC3A reactivity was detected by standard immunohistochemical staining, as described previously [35]. In brief, TMA slides were treated with a rabbit polyclonal cleaved LC3A antibody (Cat. #AP1805a, Abcepta, San Diego, CA, USA) at a 1:50 dilution for 60 min at room temperature and subsequently rinsed with Tris-buffered saline containing Tween-20 (TBS-T). The slides were then incubated for 15 min with DAKO EnVision FLEX+ Mouse LINKER (Glostrup Municipality, Glostrup, Denmark), washed again with TBS-T, and subjected to a 30-min incubation with an anti-mouse secondary antibody (Dako EnVision FLEX/HRP; DM822). Antibody staining was visualized by treating slides with EnVision FLEX DAB+ Chromogen (Dako; DM827) plus EnVision FLEX Substrate Buffer (Dako; DM823). Lastly, the slides were counterstained with hematoxylin, rinsed with cold water, and dipped 10 times in Scott’s Bluing Reagent before being washed with cold water again, dehydrated, and mounted.

It is recognized that there can be significant interobserver variability in IHC scoring [36] but one of the ways to overcome this is through reporting by specialists with particular expertise in the area. In our institution, 3 anatomical pathologists with expertise in colorectal cancer assessed the IHC scores independently. Consensus of scoring was determined through a review of 10 initial samples.

LC3A expression was quantified by multiplying the percentage of stained cells by the average staining intensity per individual cell. Staining intensity was rated on a scale from 0 to 3 as follows: 0, negative; 1, mild; 2, moderate; and 3, strong. The percentage of positive cells was also scored from 0 to 4 as follows: 0, <5%; 1, 5–25%; 2, 26–50%; 3, 51–75%; and 4, >75%. Tumor samples were sorted into two categories based on cell positivity scores: a negative expression group (score of 0) and a positive expression group (scores ranging from 1 to 4).

### 2.4. Statistical Analysis

Statistical analysis was conducted using SPSS for Windows version 29.0.2.0 (IBM Corporation, Armonk, NY, USA). Survival analysis was performed for the entire cohort, followed by further subgroup analyses utilizing tumor stage as a covariate. The prognostic implications of LC3A protein expression patterns in samples obtained from the TC and TP were evaluated by univariate and multivariate analyses, employing Kaplan–Meier survival curves and Cox’s proportional hazards modeling; hazard ratios (HRs) and 95% confidence intervals (95% CIs) were calculated. Covariates included sex, age, tumor-node-metastasis (TNM) stage, grade, LN involvement, metastasis stage at diagnosis, lymphovascular invasion (LVI), and perineural invasion (PNI), along with adjuvant and neoadjuvant therapies. The statistical significance of the findings from both univariate and multivariate analyses was assessed using the Mann–Whitney *U* test, with *p*-values < 0.05 deemed significant.

## 3. Results

### 3.1. Study Population

The characteristics of the rectal cancer patients included in this study are outlined in Table 1. The patients ranged in age from 35 to 100 years, with a median age of 72 years. The group was approximately two-thirds male (66.6%), and most (65.8%) were diagnosed at tumor stage T3 or T4. Additionally, nearly half (47.1%) of patients had LN-positive disease, with 6.5% diagnosed with metastatic disease and 7.4% having poorly differentiated tumors. Nearly one-fourth (23%) of the patients exhibited LVI, and 17% showed signs of PNI. Within this patient cohort, 21.5% received neoadjuvant therapy before surgery to shrink tumors and potentially increase the likelihood of successful treatment, and 30.4% underwent adjuvant treatment. The patients were monitored for a median duration of 3.2 years, ranging from 0.02 to 12.6 years.

### 3.2. Association Between LC3A Expression Patterns and Patient Clinicopathological Features

Tumor tissue samples from the TC and TP, as well as tumor-adjacent normal mucosa, normal mucosa further from the tumor, and the affected LNs, were subjected to immunohistochemical staining for the autophagy marker LC3A. In all samples, the TC was considered to be the area at the center of the tumor with the highest mitotic activity, whereas the TP was identified as the most mitotically active area at the outer invasive zone of each tumor.

**Figure 1:**
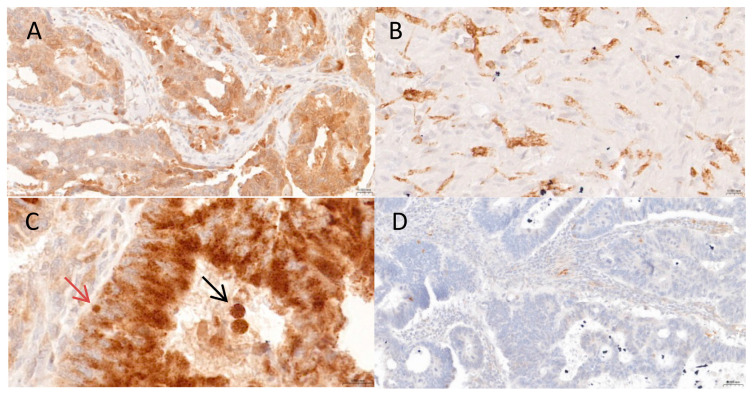
Patterns of microtubule-associated protein 1 light chain 3 alpha (LC3A) immunohistochemical staining in rectal cancer tissue. (**A**,**B**) Diffuse cytoplasmic (**A**) and perinuclear (**B**) LC3A staining patterns (magnification, 40×). (**C**) The stone-like structure LC3A staining pattern is indicative of autophagic vacuoles (arrows) in tumor tissue (magnification, 40×); note the large SLSs within luminal necrotic cells reflecting a high concentration of LC3A (black arrow) and a smaller SLS at the base of a malignant cell (red arrow). (**D**) Negative immunostaining for LC3A protein expression (magnification, 20×).

Three distinct patterns of autophagic activity were identified in rectal cancer by LC3A expression: diffuse cytoplasmic, perinuclear, and a SLS pattern (Figure 1A–C). The diffuse cytoplasmic pattern was observed as granular brown staining throughout the entire cytoplasm of cells in 4.9% of TC samples (12/243) and in 5.8% (14/243) of TP samples (Figure 1A). Perinuclear staining was visualized in the form of crescentic or ring-like perinuclear structures and detected in 23.5% (57/243) of TC and 28.4% of TP (69/243) samples (Figure 1B). Lastly, SLSs—recognized as dense, rounded cytosolic structures typically enclosed within LC3A-positive vacuoles (Figure 1C)—were detected in 4.5% (11/243) of TC samples and in 4.9% (12/243) of TP samples. Among the remaining samples, 67.9% (165/243) of TC and 63.7% (155/243) of TP samples were triple-negative for cytoplasmic, perinuclear, and SLS staining patterns and, thus, classified as LC3A-negative (Figure 1D).

We then investigated the relationship between patient clinicopathological characteristics and the three different patterns of LC3A staining, both in the TC (Table 2) and TP (Table 3). Our results show that sex (*p* = 0.046) and LN involvement (*p* = 0.028) were significantly associated with the perinuclear LC3A staining pattern in the TC, whereas the diffuse cytoplasmic and SLS staining patterns in the TC were not associated with any variables (i.e., age, tumor stage, grade, metastasis, LVI, or PNI). In contrast, an increased prevalence of the diffuse cytoplasmic pattern within the TP was correlated with tumor stage (*p* = 0.023); a trend toward linkage with LN involvement did not reach statistical significance (*p* = 0.075). Within the TP, we further found that a SLS LC3A staining pattern was associated with tumor grade *(p* < 0.001) and LVI (*p* = 0.024), whereas a perinuclear staining pattern was not linked to any variables.

### 3.3. Prognostic Implications of LC3A Expression in Rectal Cancer

**Figure 2:**
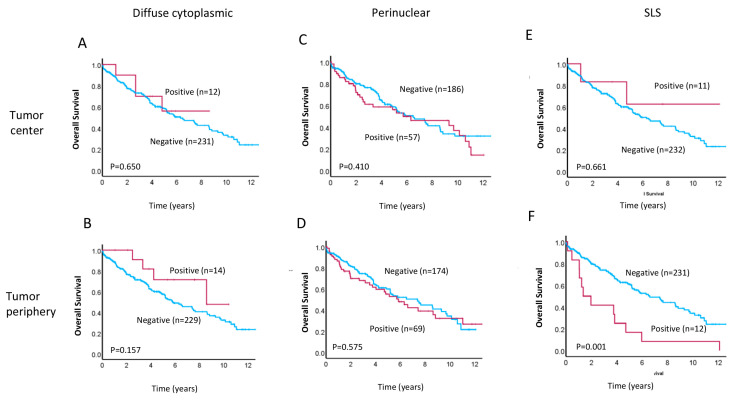
Kaplan–Meier survival analyses for overall survival in rectal cancer patients in our cohort with positivity (red lines) vs. negativity (blue lines) for the (**A**,**B**) diffuse cytoplasmic, (**C**,**D**) perinuclear, and (**E**,**F**) stone-like structure patterns of LC3A staining in the tumor center and tumor periphery. Significance was determined using the log–rank test, and *p*-values are shown.

**Figure 3:**
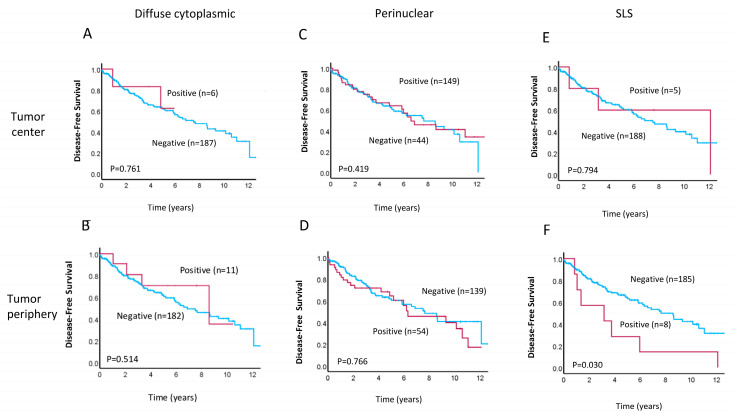
Kaplan–Meier survival analyses for disease-free survival (DFS) in rectal cancer patients with positivity (red lines) vs. negativity (blue lines) for the (**A**,**B**) diffuse cytoplasmic, (**C**,**D**) perinuclear, and (**E**,**F**) stone-like structure patterns of LC3A staining in the tumor center and tumor periphery (*n* = 193). Only patients with DFS data available were included in these analyses. Significance was determined using the log–rank test, and *p*-values are shown.

LC3A has been reported to serve as a prognostic marker for various types of cancer, with a recent meta-analysis finding that a LC3A expression pattern is indicative of prognosis in colorectal cancer patients [32]. We, therefore, explored the association between patterns of LC3A protein expression and clinical prognosis in our patient cohort. Kaplan–Meier survival analyses revealed that positivity for the SLS LC3A staining pattern in the TP was significantly associated with worse overall survival (OS; *p* = 0.001, Figure 2F) and decreased disease-free survival (DFS; *p* = 0.03, Figure 3F) relative to SLS negativity in the TP. In contrast, we did not observe any significant differences in OS or DFS for patients with positive vs. negative diffuse cytoplasmic (Figure 2A,B and Figure 3A,B) or perinuclear (Figure 2C,D and Figure 3C,D) LC3A staining in the TC or TP. Similarly, positivity for the SLS LC3A pattern in the TC was not associated with survival differences within this patient cohort (Figure 2E and Figure 3E).

We next performed univariate analysis, the results of which indicated that LC3A SLS positivity in the TP was significantly associated with reduced OS (HR = 2.712, 95% CI: 1.470–5.009, *p* = 0.001, Table 4) in our cohort of rectal cancer patients. This association between LC3A SLS positivity in the TP and reduced OS was further maintained in multivariate Cox regression analyses (HR = 2.6313, 95% CI: 1.090–6.349, *p* = 0.031). In addition, disease metastasis (HR = 2.914, 95% CI: 1.103–7.696, *p* = 0.031) and PNI (HR = 1.997, 95% CI: 1.035–3.893, *p* = 0.042) remained significantly associated with worse OS in multivariate analyses (Table 4). These data suggest that a SLS pattern of LC3A expression, together with disease metastasis and PNI, represent strong prognostic factors for worse OS in patients with rectal cancer.

### 3.4. The SLS Pattern as Putative Prognostic Factor for Aggressive Tumors

**Figure 4:**
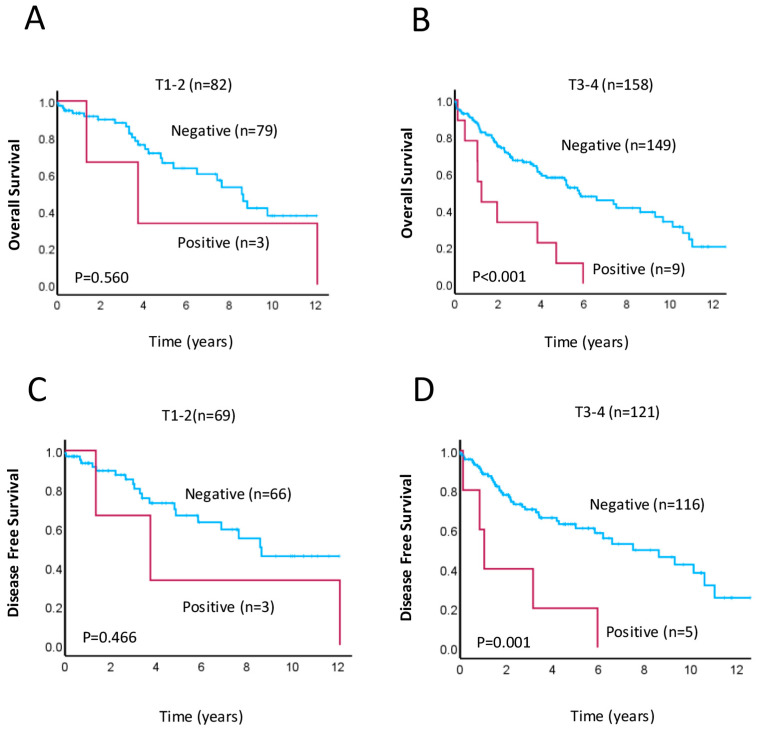
Kaplan–Meier survival analyses for patients grouped by tumor stage, with positive (red lines) vs. negative (blue lines) stone-like structure LC3A staining in the tumor periphery. (**A**,**B**) Overall survival and (**C**,**D**) disease-free in patients with early-stage (T1–T2; **A**,**C**) and advanced-stage (T3–T4; **B**,**D**) disease. Significance was determined using the log–rank test, and *p*-values are shown.

Lastly, given our finding that rectal cancer patients with positive SLS LC3A staining in the TP have worse OS than patients with negative for this staining pattern, we performed Kaplan–Meier survival analysis for patients who were grouped by tumor stage (T1–T2 vs. T3–T4). The results showed that SLS positivity in the TP was associated with decreased OS (*p* < 0.001, Figure 4B) and reduced DFS (*p* = 0.001, Figure 4D) only for those in the T3–T4 subgroup, suggesting that a TP-specific SLS pattern for LC3A staining may be a useful prognostic biomarker for rectal cancer patients with advanced disease. Additional Cox regression analysis confirmed that a SLS LC3A staining pattern within the TP in patients from the T3–T4 subgroup was significantly associated with worse OS (HR = 3.347, 95% CI: 1.657–6.760, *p* = 0.001; Table 4).

## 4. Discussion

Rectal tumors account for an increasing proportion of CRC cases, particularly those diagnosed in younger patients [2]. Although several candidate biomarkers and prognostic indicators for this disease have been identified, our understanding of the factors that modulate rectal cancer outcomes remains incomplete [5,6,7,8,9,10,11,12,13,14]. Here, we investigated the prognostic significance of distinct patterns of immunohistochemical staining for the autophagy marker LC3A in central and peripheral tumor tissue from 243 rectal cancer patients. The results showed that a SLS pattern of LC3A staining in the TP, but not the TC, was associated with worse OS and DFS, specifically for patients with more advanced rectal cancer tumors, that is, those in the T3–T4 subgroup.

Autophagy is a cellular self-degradation process required for both the baseline turnover of cellular components and for promoting cell survival in response to conditions such as starvation and genomic damage [21,22]. Two types of autophagy are conserved in eukaryotes: macroautophagy and microautophagy. Macroautophagy is a more elaborate pathway involving the formation of new membrane structures, while microautophagy is a simpler process of direct lysosomal engulfment. Given these complex roles, autophagy has been described as a “double-edged sword” in cancer, functioning to either inhibit or promote oncogenesis, dependent on the stage of cancer development and environmental context [18]. Accordingly, a wealth of prior studies investigating the role of autophagy in CRC and rectal cancer have reported conflicting results regarding whether it primarily functions as a tumor suppressor or oncogenic factor in these diseases and how it may affect therapy outcomes [29,30,37].

The biological active zone at the periphery of a tumor is characterized by upregulated proteins involved in tumorigenic signaling nodes, possesses a unique cellular milieu, and plays a crucial role in tumor invasion and metastasis, with cells actively breaking through the basal membrane and migrating into surrounding tissues [38]. 

The tumor periphery therefore experiences a complex interplay of mechanical and biochemical stressors that influence tumor growth, progression, and response to treatment. Stone-like structures are hypothesized to appear under conditions of extreme stress [39], where an abnormal autophagic response can result in overload of degenerative cytoplasmic components, depletion of enzymes or lysosomal enzyme defects, and ensuing accumulation of debris and massive destruction of tumor cells. The association of the SLS pattern of LC3A staining in the TP with tumor grade and lymphovascular invasion in our study lends support to this hypothesis.

In the present study, we focused specifically on expression patterns of LC3A in rectal tumors, finding that the presence of a punctate SLS pattern of staining in the TP was associated with poor survival outcomes. LC3 proteins are ubiquitin-like molecules that are commonly used as markers for active autophagy due to their localization within the inner and outer autophagosomal membrane [15,40,41]. Altered expression of LC3A/B has been reported in numerous cancers, including pancreatic, breast, and gastrointestinal cancers [18,19]. Studies measuring LC3 expression in tissue from CRC and rectal cancer patients have generally reported an association between elevated protein levels and improved survival outcomes [31,32,33]. However, correlations between elevated LC3 expression and adverse outcomes, including poor response to therapy, have also been noted [42,43]. Intriguingly, one study analyzed LC3B expression together with that of p62, another autophagosomal marker, and found that a high LC3B “dot-like” pattern combined with a low p62 “dot-like” pattern was associated with poor survival, suggesting that activated autophagy may lead to worse outcomes in CRC [43]. Unlike in our study, however, they did not observe differences in staining within the TP vs. the TC.

Consistent with our present findings, Giatromanolaki et al. observed three patterns of LC3A staining in CRC tumors and found the SLS pattern to be associated with metastasis and poor prognosis in CRC [34]. Unlike in our study, however, they further noted a correlation between the perinuclear staining pattern and improved survival outcomes. The authors postulated that the SLS pattern may be indicative of an abnormal or excessive autophagic response, whereas the perinuclear pattern reflects normal baseline autophagic activity [34]. It should be pointed out that Giatromanolaki et al. did not draw any distinction between rectal cancer and colon cancer in their analysis. We know that, due to clear differences in molecular carcinogenesis, pathology, and multimodal treatment, rectal cancer and colon cancer should be regarded as distinct clinical entities [44]. A strength of our study is that all cancers included in the study were rectal cancers.

Within the tumor microenvironment, different regions exhibit variable levels of oxygen deprivation, with the most acute hypoxia present within the TC [45]. This hypoxic environment within the tumor can induce stress response pathways, including autophagy, to promote cancer cell survival [15,18,19,41]. Our observation that the SLS pattern of LC3A expression is associated with poor OS and DFS only when observed in the TP suggests that differences in oxidative and replicative stress conditions between the TC and TP may be one factor underlying the TP-specific correlation with LC3A expression. Of note, hypoxia-induced autophagy within the tumor microenvironment has been reported to induce epithelial-to-mesenchymal transition to promote metastasis of CRC cells [29], and in one study, LC3B expression specifically at the TP was associated with clinicopathological features associated with tumor aggressiveness [46]. Thus, we speculate that the SLS pattern of LC3A staining observed in our study may be indicative of increased metastatic potential, ultimately leading to worse patient outcomes. Consistent with this interpretation, the SLS pattern of LC3A staining, albeit not specifically at the TP, was found to be associated with the development of distant metastases in CRC [34]. There is clinical value in our prognostic data, but more work is needed before the predictive value of the autophagic patterns described in rectal cancers is fully appreciated.

This study has several limitations. In particular, we detected only a small number of patients with SLS positivity in the TC or TP, particularly among those with aggressive disease (i.e., T3–T4 subgroup), highlighting the need for additional studies assessing the prognostic implications of LC3A expression patterns in larger cohorts of rectal cancer patients. We further note that LC3A staining may reflect elevated autophagy or the inhibition of LC3A degradation [15]. Therefore, mechanistic analyses are needed to confirm an activation of autophagy within the TP of rectal cancer tumors. One key limitation of this study using tissue microarray (TMA) technology is that it only examines a small, representative sample of the tissue, not the entire slide. Moreover, the precise functional implications of the SLS LC3A staining pattern relative to the other patterns observed in this study remain unclear. We also did not measure expression of other autophagy proteins, such as p62 and Beclin-1, which were previously reported to have prognostic value for colon and rectal cancers [29,30,37]. All 51 patients with neoadjuvant therapy were included in the analyses. Our central database did not contain information about whether these patients had a complete clinical response given that they were referred from multiple private surgeons and different hospitals.

Lastly, although we have focused on autophagy (i.e., macroautophagy), our findings may also indicate a link between rectal cancer and mitophagy, given that LC3A functions in both processes, and notably, recent findings identified a mitophagy-related gene signature associated with CRC prognosis [47].

## 5. Conclusions

The role of autophagy in cancers of the colon and rectum is complex and not fully understood. In the present study, we aimed to determine the prognostic value of the autophagy marker LC3A in rectal cancer tumors. Our immunohistochemical analysis of TC and TC samples and adjacent healthy tissue from 243 patients revealed that a SLS pattern of LC3A expression within the TP was associated with worse OS and DFS in our overall cohort and specifically in advanced-stage patients (T3–T4). These findings suggest that TP-specific SLS LC3A staining in rectal cancer may be a useful prognostic biomarker for poor survival outcomes in rectal cancer patients with advanced disease. Our results further imply that elucidating the prognostic implications of LC3A (and potentially other autophagy proteins) in rectal cancer is dependent on determining the specific pattern and location of expression within tumor tissue.

## Figures and Tables

**Table 1:** Characteristics of the patients included in this study (*n* = 243).

**Characteristics**	**Patients (*n* = 243)**	**%**
Age median	72 (35–100 years)	
<72	113	46.5
≥72	130	53.5
Sex		
Male	161	66.6
Female	83	33.4
Tumor stage		
T1, T2	82	34.2
T3, T4	158	65.8
Node stage		
N0	126	52.9
N1, N2	112	47.1
Metastasis stage		
M0	215	93.5
M1	15	6.5
Grade		
1, 2	225	92.6
3	18	7.4
Lymphovascular invasion		
Absent	185	76.8
Present	56	23.2
Perineural invasion		
Absent	200	83
Present	41	17
Treatment		
Neoadjuvant therapy		
No	186	78.5
Yes	51	21.5
Adjuvant therapy		
No	155	69.6
Yes	66	30.4

**Table 2:** Associations between LC3A expression in the tumor center and patient clinico-histopathological data (*n* = 243).

		**Diffuse Cytoplasmic**			**Perinuclear**			**SLS**		
		**Negative (%)**	**Positive (%)**	***p*-Value**	**Negative (%)**	**Positive (%)**	***p*-Value**	**Negative (%)**	**Positive (%)**	***p*-Value**
Sex	Male	66.7	58.3	0.552	62.9	77.2	0.046	66.8	54.5	0.401
	Female	33.3	41.7		37.1	22.8		33.2	45.5	
Age	<72	45.5	66.7	0.151	48.9	38.6	0.171	45.7	63.6	0.244
	≥72	54.5	33.3		51.1	61.4		54.3	36.4	
Tumor stage	T1–T2	33.3	34.2	0.235	34.9	31.5	0.636	33.2	45.5	0.145
	T3–T4	66.7	65.8		65.1	68.5		66.8	54.5	
Node stage	Negative	51.8	75	0.116	56.8	39.6	0.028	52.0	72.7	0.178
	Positive	48.2	25		43.2	60.4		48.0	27.3	
Metastasis stage	M0	93.6	90	0.629	93.2	94.4	0.742	93.7	88.9	0.569
	M1	6.4	10		6.8	5.6		6.3	11.1	
Grade	1, 2	92.2	100	0.315	94.1	87.7	0.108	92.2	100	0.337
	3	7.8	0		5.9	12.3		7.8	0	
Lymphovascular invasion	Absent	76	91.7	0.21	77.4	74.5	0.658	76.1	90.9	0.255
	Present	24	8.3		22.6	25.5		23.9	9.1	
Perineural invasion	Absent	82.5	91.7	0.412	83.9	80	0.502	82.6	90.9	0.474
	Present	17.5	8.3		16.1	20		17.4	9.1	
Adjuvant therapy	No	68.9	81.8	0.365	70.6	66	0.541	69.1	80	0.464
	Yes	31.1	18.2		29.4	34		30.9	20	
Neoadjuvant therapy	No	79.1	66.7	0.307	78.9	77.2	0.786	79.2	63.6	0.22
	Yes	20.9	33.3		21.1	22.8		20.8	36.4	

**Table 3:** Associations between LC3A expression in the tumor periphery and patient clinico-histopathological data (*n* = 243).

		**Diffuse Cytoplasmic**			**Perinuclear**			**SLS**		
		**Negative (%)**	**Positive (%)**	***p*-Value**	**Negative (%)**	**Positive (%)**	***p*-Value**	**Negative (%)**	**Positive (%)**	***p*-Value**
Sex	Male	66.8	57.1	0.458	66.3	66.2	0.987	66.7	58.3	0.552
	Female	33.2	42.9		33.7	33.8		33.3	41.7	
Age	<72	46.7	42.9	0.771	45.1	50	0.496	45.7	41.7	0.731
	≥72	53.3	57.1		54.9	50		54.3	58.3	
Tumor stage	T1–T2	32.6	61.5	0.023	36.4	28.4	0.238	34.5	25	0.492
	T3–T4	67.4	38.5		63.6	71.6		65.5	75	
Node stage	Negative	51.6	76.9	0.075	55.2	47	0.253	54.0	33.6	0.163
	Positive	48.2	23.1		44.8	53		46.0	66.4	
Metastasis stage	M0	93.1	100	0.347	92.2	96.9	0.195	94.0	83.3	0.144
	M1	6.9	0		7.8	3.1		6.0	16.7	
Grade	1, 2	92.1	100	0.276	93.1	91.2	0.599	93.9	66.7	<0.001
	3	7.9	0		6.9	8.8		6.1	33.3	
Lymphovascular invasion	Absent	76.3	84.7	0.491	77.5	75	0.684	72.8	50	0.024
	Present	23.7	15.3		22.5	25		7.2	50	
Perineural invasion	Absent	82.5	92.3	0.358	83.2	82.4	0.869	83.8	66.7	0.123
	Present	17.5	7.7		16.8	17.6		16.2	33.3	
Adjuvant therapy	No	69.5	71.4	0.877	72.2	62.7	0.179	70.3	50	0.22
	Yes	30.5	28.6		27.8	37.3		29.7	50	
Neoadjuvant therapy	No	77.6	92.9	0.177	82.8	67.6	0.161	77.9	90.1	0.304
	Yes	22.4	7.1		17.2	32.4		22.1	9.1	

**Table 4:** Cox regression analyses assessing the association between overall survival and clinicopathological features, including LC3A expression, in rectal cancer patients (*n* = 243).

		**Univariate**			**Multivariate**		
	***n* (%)**	**HR**	**95% CI**	***p*-Value**	**HR**	**95%**	***p*-Value**
SLS TP *							
Positive	4.9	2.712	1.470–5.009	0.001	2.6313	1.090–6.349	0.031
Negative	95.1						
Sex							
Male	66.6	1.03	0.693–1.530	0.885	1.127	0.697–1.822	0.685
Female	33.4						
Age							
<72	46.5	1.416	0.954–2.101	0.084	1.271	0.770–2.100	0.349
≥72	53.5						
Tumor stage							
T1, T2	34.2	1.426	1.047–2.460	0.03	1.426	0.842–2.415	0.187
T3, T4	65.8						
Node stage							
Negative	52.9	1.405	0.956–2.064	0.083	1.489	0.891–2.489	0.128
Positive	47.1						
Metastasis stage							
M0	93.5	5.102	2.699–9.643	<0.001	2.914	1.103–7.696	0.031
M1	6.5						
Grade							
1, 2	92.6	1.719	0.942–3.135	0.077	1.343	0.252–1.763	0.414
3	7.4						
Lymphovascular invasion							
Absent	76.8	2.011	1.333–3.033	0.001	1.688	0.919–3.101	0.092
Present	23.2						
Perineural invasion							
Absent	83	2.478	1.602–3.833	<0.001	1.997	1.035–3.893	0.042
Present	17						
Adjuvant therapy							
No	78.5	0.567	0.344–0.934	0.026	0.306	0.163–0.577	0.065
Yes	21.5						
Neoadjuvant therapy							
No	69.6	0.984	0.614–1.576	0.945	1.254	0.672–2.340	0.476
Yes	30.4						
T1–T2 SLS	51.2				1.529	0.361–6.472	0.564
T3–T4 SLS	48.8				3.347	1.657–6.760	0.001

CI, confidence interval; HR, hazard ratio; SLS, stone-like structure; * TP, tumor periphery.

## Data Availability

The data presented in this study are available on request from the corresponding author. The data are not publicly available due to data size and privacy.

## Abbreviations

The following abbreviations are used in this manuscript:

CI	Confidence interval
CRC	Colorectal cancer
DFS	Disease-free survival
HR	Hazard ratio
LN	Lymph node
LVI	Lymphovascular invasion
MDPI	Multidisciplinary Digital Publishing Institute
OS	Overall survival
PNI	Perineural invasion
SLS	Stone-like structure
TC	Tumor center
TMA	Tissue microarray
TNM	Tumor-node-metastasis
TP	Tumor periphery
